# Integrated transcriptomics and metabolomics study of embryonic breast muscle of Jiaji ducks

**DOI:** 10.1186/s12864-024-10452-6

**Published:** 2024-06-01

**Authors:** Lihong Gu, Jile Chen, Chengjun Hu, Dingfa Wang, Shuqian Huan, Guang Rong, Renlong Lv, Tieshan Xu

**Affiliations:** 1 Tropical Crop Genetic Resource Research Institute, Chinese Academy of Tropical Agricultural Sciences, Haikou, 571101 Hainan P.R. China; 2 Institute of Animal Science and Veterinary Medicine, Hainan Academy of Agricultural Sciences, Haikou, 571100 P.R. China; 3 School of Animal Science and Technology, School of Animal Medicine, Huazhong Agricultural University, Hubei Province, Hongshan District, Wuhan, 430072 China; 4 College of Animal Science, Hainan University, Haikou, 570228 P.R. China

**Keywords:** Jiaji duck, Transcriptome, Metabolome, Breast muscle, Joint analysis

## Abstract

Because number of matured muscle fibers in poultry does not increase after birth, the meat yield is mainly determined during embryogenesis. We previously indicated breast muscle grew rapidly from 18th day after hatching (E18) to E27, and almost stopped from E27 to E34 of Jiaji ducks, while the mechanism is unclear. This study utilized RNA-seq to explore the related genes of muscle development and their relationship with small molecule metabolites at E18, E27 and E34 of Jiaji ducks. Several thousand differentially expressed genes (DEGs) were detected among E18, E27 and E34. DEGs expression profiles included 8 trend maps, among which trend 1 was opposite to and trend 6 was consistent with breast muscle development trend of Jiaji ducks. Through joint analysis between trend 1 of DEGs and trend 1 of differential metabolites (DEMs), protein digestion and absorption pathway stood out. The decrease of *COL8A2* gene expression will lead to the decrease of arginine content, which will inhibit the development of breast muscle in embryonic Jiaji duck. Similarly, joint analysis between trend 6 of DEGs and trend 6 of DEMs indicated the increase of *GAMT* gene expression will cause the increase of proline content, and then promote the development of breast muscle of Jiaji duck in embryonic period. These results will be helpful for further understanding the mechanism of muscle yields of Jiaji ducks.

**Supplementary Information:**

The online version contains supplementary material available at 10.1186/s12864-024-10452-6.

## Introduction

Poultry is one of the most important meat resources in the world, providing a variety of products for consumers, such as high-quality meat, eggs, eiderdown and so on. To meet consumer’s demand for animal products, the breeding, feeding and slaughtering technologies of traditional meat poultry such as broilers and meat ducks have been continuously innovated to improve the production efficiency and the quality of products of poultry. As an excellent meat type poultry, Muscovy duck (*Cairina moschata*) is very popular because of its fast growth, delicious meat and higher intramuscular fat [1–3].

Poultry skeletal muscle development is a complex process, affected by genetic, nutritional and other factors [4, 5]. Skeletal muscle is made up of thousands of muscle cells, which have different sizes and shapes and are arranged in a specific way on the bone. During skeletal muscle development, mesodermal progenitor cells are selected to form myoblasts, a process known as muscle development or muscle formation. Myoblasts further fuse to form multinucleated myotubes, and myotubes further differentiate to form muscle fibers with contraction characteristics [6]. Since the number of muscle fibers in most animals does not increase after birth but is determined at the embryonic stage, muscle production in adult animals is determined during embryogenesis [7].

Transcriptome sequencing is a basic method to study the genes and functions related to muscle development in livestock and poultry, and has been widely used. Feng et al. found key genes related to porcine skeletal muscle development through transcriptome analysis [8]. Li et al. revealed the changes in gene expression between wild chickens and domestic chickens induced by domestication through transcriptome analysis of Gastrocnemius [9]. Hu et al. integrated transcriptomics and non-targeted metabolomics of Peking ducks, revealing the potential mechanism of skeletal muscle development in Peking ducks [10]. However, the proliferation and development of Jiaji duck breast muscle and its relationship with small molecule metabolites have not been studied.

Jiaji duck is a kind of Muscovy duck, which is native to Jiaji Town, Qionghai City, Hainan Province, China. Jianjiaji ducks have excellent qualities such as fast growth, short feeding cycle, rough feeding resistance, simple production equipment, strong disease resistance, high survival rate and high lean meat rate. Our previous study found that breast muscle cells of Jiaji ducks were mainly proliferated and the breast muscle weight increased rapidly E18 to E27. From E27 to E34, breast muscle cells almost stopped proliferating, and breast muscle weight basically stagnated (unpublished data). At the same time, we also carried out metabolomics analysis of E18, E27 and E34, and obtained a large number of small molecule metabolites related to the development and proliferation of Jiaji duck breast muscle (unpublished data). However, the genetic basis of muscle development and proliferation in Jiaji duck embryos and its relationship with metabolic small molecules are still unclear. Therefore, RNA-seq technology was used to explore the gene expression patterns, possible functions and their relationship with small molecule metabolites in the breast muscle of Jiaji ducks at E18, E27 and E34. The results of this paper will provide data support for further exploring the mechanism of proliferation and development of breast muscle for Jiaji duck and its relationship with small molecule metabolites.

## Materials and methods

### Selection of samples

The duck eggs used in this paper were obtained from Hainan Chuanwei Muscovy Duck Breeding Co., Ltd. A total of 200 eggs were selected with 81 g ± 5 g. All eggs were incubated at 37℃±0.5℃ temperature and 86–87% humidity under the same conditions. Eight eggs were selected at E18, E27 and E34, respectively. The embryos were removed from the eggs, the entire embryo was weighed, one side of breast muscle were removed and weighed, and the other side of breast muscle were also removed and were snap-frozen in liquid nitrogen and stored at − 80℃.

### RNA extraction, library construction and sequencing

Total RNA was extracted from 8 samples at each embryonic stage using TRIzol (Invitrogen, Carlsbad, CA, USA) according to the manufacturer’s protocol. The purity and concentration of RNA were verified by NanoDrop ND-1000 (NanoDrop, Wilmington, DE, USA) and agarose gel electrophoresis. The integrity of the RNA samples was evaluated using Bioanalyzer 2100 (Agilent, CA, USA) to ensure that the RIN value was greater than 0.7. About 1 ug total RNA was extracted and purified by Dynabeads Oligo (dT) 25-61005 (Thermo Fisher, CA, USA), and then the purified RNA was fragmented into small fragments by Magnesium RNA Fragmentation Module (NEB, cat.e6150, USA). Subsequently, the fragmented RNA was transcribed into complementary cDNA using reverse transcriptase. Then, with the help of E.coli DNA polymerase I and RNase H, double-strand synthesis was performed to convert these RNA-DNA complexes into DNA double-strand structures. At the same time, dUTP solution was introduced into the synthesized DNA double strands to complete the end of the double-stranded DNA to the flat end. In order to allow it to be connected to an adaptor with a terminal T base, we added an A base at each end of it. Subsequently, we used magnetic beads to screen and purify its fragment size. The double strands were digested by UDG enzyme and amplified by PCR to control the size of the library fragment in the range of 300 bp ± 50 bp. RNA sequencing was performed in Hangzhou Lianchuan Biotechnology Co., Ltd., using Novaseq6000 sequencing platform, and the sequencing strategy was PE150 (150 bp double-end sequencing).

### Bioinformatics analysis of RNA-seq

We used Cutadapt software (https://cutadapt.readthedocs.io/en/stable/, version 1.9) to remove reads containing junction contamination, low-quality bases, and default parameter uncertain bases. Then use Cutadapt to verify the sequence quality. We used HISAT2 (https://ccb.jhu.edu/software/hisat2, version 2–2.0.4) to align sequencing data to the Muscovy duck reference genome (KizCaiMos1.0) [11]. StringTie software was used to perform initial assembly of genes or transcripts, combined the initial assembly results of all samples, and used gffcompare software (http://ccb.jhu.edu/software/stringtie/gffcompare.shtml, version 0.9.8) to detect the comparison of transcripts with reference annotations to obtain the final assembly annotation results. FPKM (FPKM=[total_exon_fragments/mapped_reads(millions)×exon_length(kB)]) method was used to quantify the basic expression of all genes. The R package edgeR (https://bioconductor.org/packages/release/bioc/html/edgeR.html) was used to analyze the significant differences between the samples. The genes with FC > 2 times or FC < 0.5 times and *p* value < 0.05 were defined as differential genes, and GO and KEGG enrichment analysis were performed.

### Trend analysis

We used all DEGs obtained in this study to build a database according to the data requirements in the Lianchuan biological cloud platform through Excel software. After the database was completed, the data was analyzed through the STEM [12] cloud tool in the cloud platform to obtain the trend map of DEGs.

### Conjoint analysis

We first selected the trend subgraphs that were consistent with (transcriptome trend 6) or opposite to (transcriptome trend 1) the trend of breast muscle development or myogenic cell proliferation from the trend diagram. Secondly, the required DEGs and related metabolites were used to construct the database through Excel, and then the Veen cloud tool in the Lianchuan biological cloud platform was used to process the data to obtain the pathways enriched by DEGs and metabolites. The correlation between DEGs and related metabolites was analyzed by using the correlation clustering marker heat map cloud tool.

## Results

### Overall evaluation of RNA-Seq data

The statistical information of RNA-seq data was presented in Table 1. From Table 1, it can be seen that the sequencing of breast muscle tissue produced an average of about 42.29 million raw reads per sample, with an average base of 6.34G. After filtering, the number of valid reads ranged from 31.90 million to 49.12 million, with an average base of 6.10 G, and the percentage of valid reads was all larger than 94.22%. The Q20 and Q30 for each sample ranged from 99.96 to 99.97% and 97.89–98.38%, respectively. The results of valid data alignment to the duck reference genome were shown in Table S1 and Fig. S1. The results showed that the average ratio of reads mapped to the reference genome of E18, E27 and E34 samples were 68.19%, 65.88% and 63.15%, respectively. On average, about 80% of the reads in each sample were mapped to exons, and a small portion was mapped to gaps and introns.

**Table 1:** Statistical information of RNA-seq data

Sample	Raw Data		Valid Data		Valid Ratio(%)	Q20%	Q30%	GC content%
	Read	Base	Read	Base				
E18_1	51,318,794	7.70G	49,120,376	7.37G	95.72	99.96	98.02	48
E18_2	44,207,906	6.63G	42,637,844	6.40G	96.45	99.96	98.02	49
E18_3	41,625,394	6.24G	40,153,908	6.02G	96.46	99.96	98.00	50
E18_4	49,032,896	7.35G	47,004,982	7.05G	95.86	99.96	98.11	50
E18_5	34,816,218	5.22G	33,078,276	4.96G	95.01	99.97	98.38	49.50
E18_6	49,254,862	7.39G	47,081,622	7.06G	95.59	99.96	97.92	49
E18_7	37,277,092	5.59G	35,727,488	5.36G	95.84	99.96	97.91	50
E18_8	39,538,166	5.93G	38,019,306	5.70G	96.16	99.96	98.01	50
E27_1	44,807,248	6.72G	42,896,632	6.43G	95.74	99.96	97.89	50
E27_2	36,014,390	5.40G	33,931,090	5.09G	94.22	99.96	98.20	50
E27_3	50,835,772	7.63G	48,909,148	7.34G	96.21	99.96	97.89	50
E27_4	44,834,822	6.73G	42,701,490	6.41G	95.24	99.96	97.89	48.50
E27_5	39,579,520	5.94G	38,009,302	5.70G	96.03	99.97	98.27	49.50
E27_6	34,551,988	5.18G	33,392,038	5.01G	96.64	99.96	98.26	50
E27_7	42,407,282	6.36G	40,967,798	6.15G	96.61	99.97	98.28	50
E27_8	32,932,344	4.94G	31,900,926	4.79G	96.87	99.97	98.14	51
E34_1	38,982,814	5.85G	37,819,382	5.67G	97.02	99.97	98.19	50
E34_2	37,518,296	5.63G	36,292,284	5.44G	96.73	99.97	98.19	51
E34_3	45,707,548	6.86G	43,882,080	6.58G	96.01	99.97	98.23	50.50
E34_4	46,292,640	6.94G	44,547,798	6.68G	96.23	99.96	97.99	50.50
E34_5	48,190,280	7.23G	46,550,614	6.98G	96.60	99.97	98.07	50
E34_6	43,540,714	6.53G	42,164,096	6.32G	96.84	99.96	98.04	51
E34_7	45,208,206	6.78G	43,728,714	6.56G	96.73	99.96	98.06	51
E34_8	36,413,306	5.46G	35,162,300	5.27G	96.56	99.96	97.94	51

### DEGs analysis

**Fig. 1:**
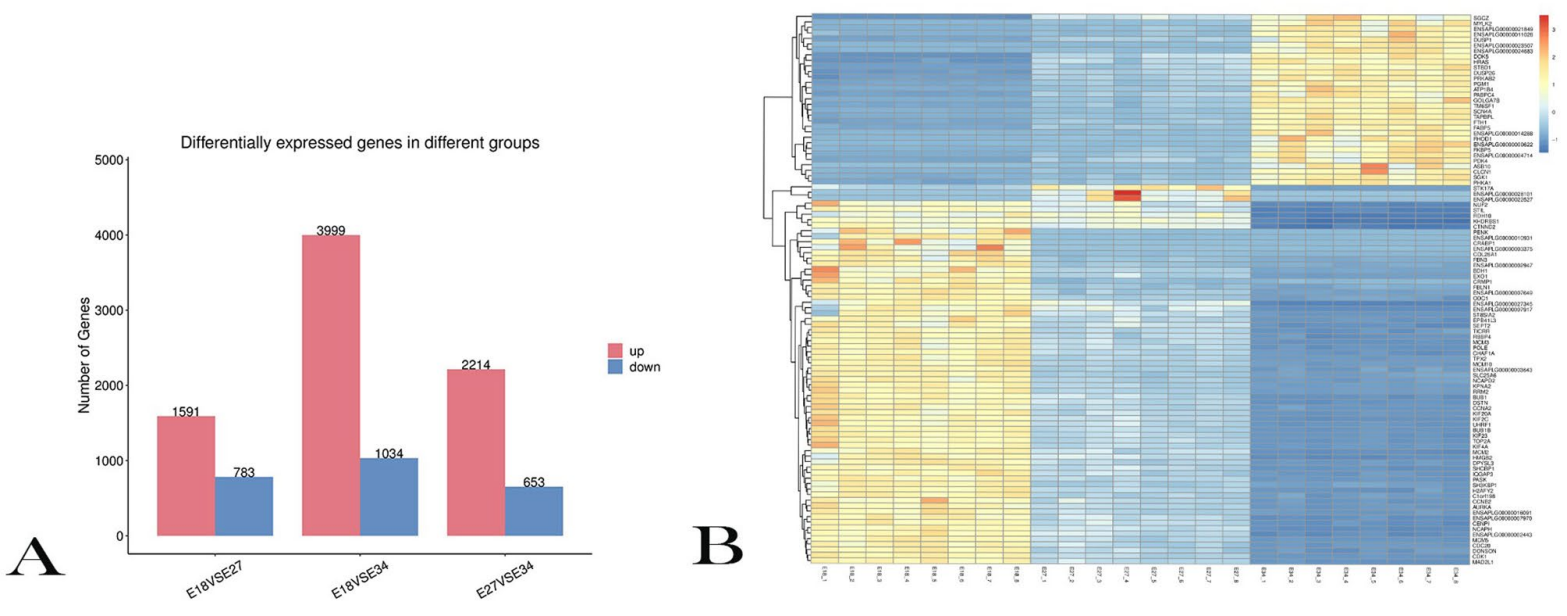
Analysis of differentially expressed genes (DEGs) (**A**) Count of DEGs in each group; (**B**) Cluster analysis of DEGs

A total of 9,855 genes were detected among the three periods of Jiaji duck embryo breast muscle tissues, accounting for 62% of the total number of Muscovy duck annotated genes ^[11]^. Through differential expression analysis, 2,374 DEGs were detected for E18 VS E27 with 1,591 up-regulated and 783 down-regulated (Table S2), 5,033 DEGs were detected for E18 VS E34 with 3,999 up-regulated and 1,034 down-regulated (Table S3), 2,867 DEGs were detected for E27 VS E34 with 2,214 up-regulated and 653 down-regulated (Table S4, Fig. 1A). Through heat map analysis, we found that the gene expression levels were different at different stages (Fig. 1B), which showed that there were significant differences between the samples at different stages.

**Fig. 2:**
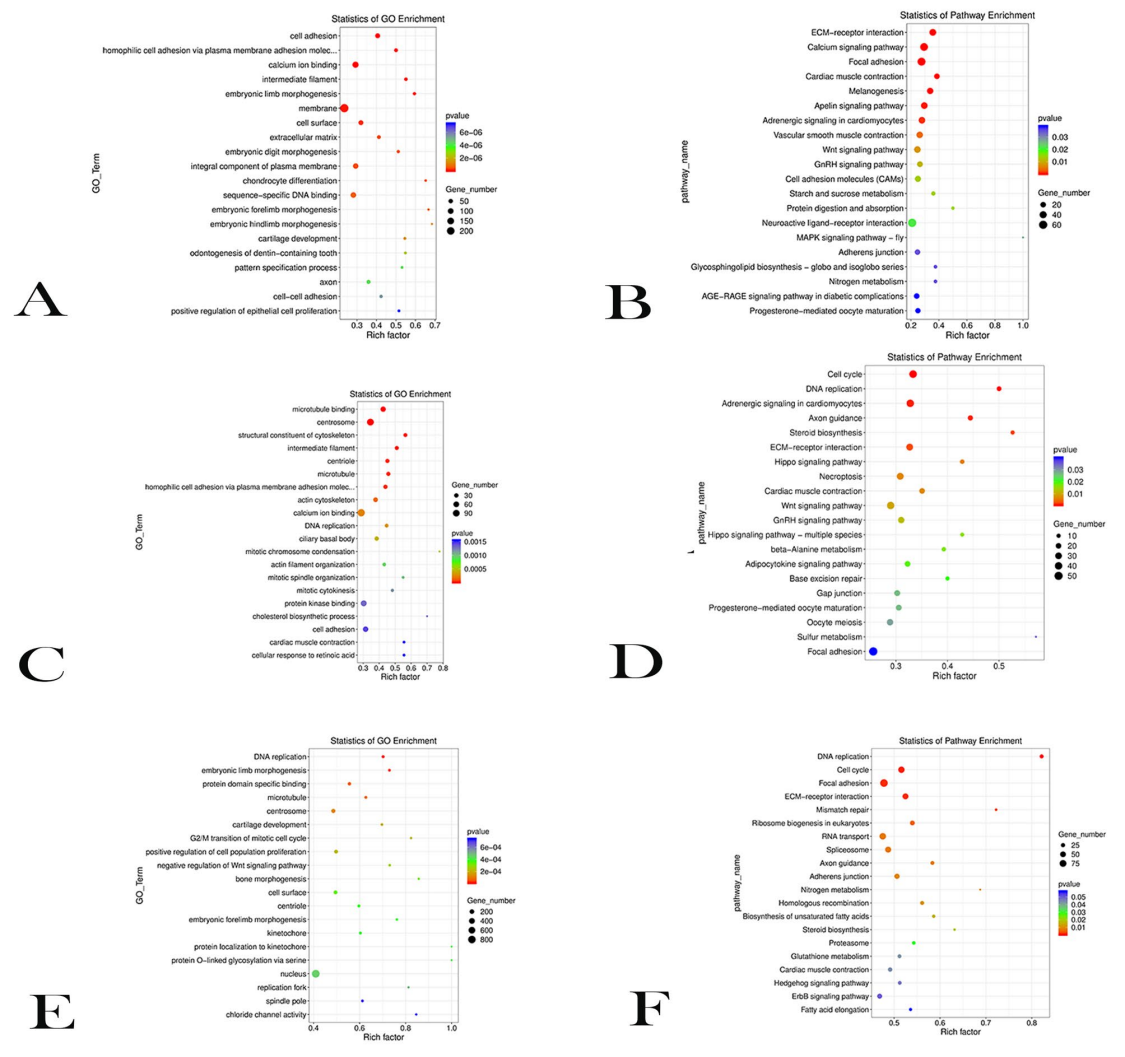
GO and KEGG analysis of DEGs in E18 VS E27, E27 VS E34, E18 VS E34. (**A**) GO biological processes enriched by DEGs between E18 VS E27. (**B**) KEGG pathways enriched by DEGs between E18 VS E27. (**C**) GO biological processes enriched by DEGs between E27 VS E34. (**D**) KEGG pathways enriched by DEGs between E27 VS E34. (**E**) GO biological processes enriched by DEGs between E18 VS E34. (**F**) KEGG pathways enriched by DEGs between E18 VS E34

For DEGs between E18 VS E27, E18 VS E34, and E27 VS E34, GO enrichment and KEGG pathway analysis was performed and the top 20 GO biological processes and KEGG pathways were selected to construct rich factor figures. For DEGs in E18 VS E27, the GO terms of cell adhesion, homophilic cell adhesion via plasma membrane adhesion molecules, calcium ion binding, and chondrocyte differentiation were significantly enriched (*P* < 0.05). Cell adhesion, extracellular matrix and chondrocyte differentiation were related to cell proliferation and differentiation (Fig. 2A). KEGG pathway analysis showed that DEGs were mainly enriched in ECM-receptor interaction, Calcium signaling pathway, Focal adhesion, MAPK signaling pathway, and Wnt signaling pathway (*P* < 0.05, Fig. 2B). ECM-receptor interaction, MAPK signaling pathway, Calcium signaling pathway, Apelin signaling pathway, Wnt signaling pathway were related to cell proliferation and differentiation.

For DEGs in E27 VS E34, the GO terms of microtubule binding, centrosome, and structural constituent of cytoskeleton were significantly enriched (*P* < 0.05). In these 20 biological processes, mitotic cytokinesis, cholesterol biosynthetic process, cell adhesion, cellular response to retinoic acid were associated with cell proliferation and differentiation (Fig. 2C). KEGG pathway analysis showed that DEGs were mainly enriched in Cell cycle, ECM − receptor interaction, Adrenergic signaling in cardiomyocytes, Wnt signaling pathway, Focal adhesion and other signaling pathways (*P* < 0.05, Fig. 2D). Among them, ECM − receptor interaction, Focal adhesion, Cardiac muscle contraction, Wnt signaling pathway, GnRH signaling pathway, Progesterone − mediated oocyte maturation were the shared pathways enriched by DEGs in E18 VS E27. Most of these pathways were related to cell proliferation and differentiation.

For DEGs in E18 VS E34, the GO terms of DNA replication, embryonic limb morphogenesis, protein domain specific binding, and cell surface were significantly enriched (*P* < 0.05, Fig. 2E). In the enriched biological processes, the terms of G2 / M transition of mitotic cell cycle, positive regulation of cell population proliferation, chloride channel activity were related to proliferation and differentiation. KEGG pathway analysis showed that DEGs were mainly enriched in Cell cycle, Focal adhesion, ECM − receptor interaction, and ErbB signaling pathway (*P* < 0.05, Fig. 2F). ECM − receptor interaction, ErbB signaling pathway and Hedgehog signaling pathway were associated with cell proliferation and differentiation.

### The expression profile analysis of DEGs

**Fig. 3:**
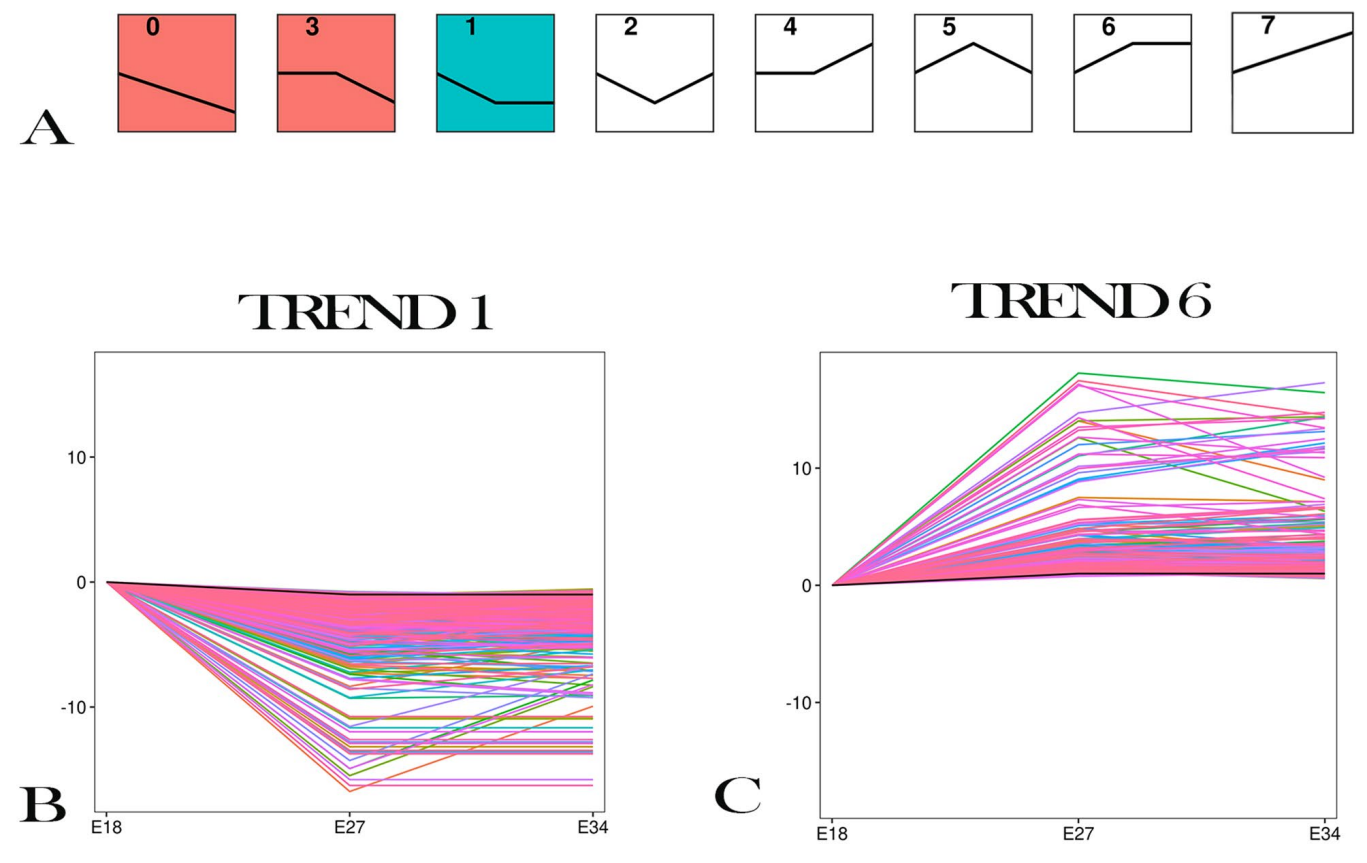
The expression profiles of DEGs. (**A**) The expression profiles of DEGs. (**B**) The expression profiles of DEGs in trend 1. (**C**) The expression profiles of DEGs in trend 6

Through our previous results, the weight of breast muscle in Jiaji duck embryo grew rapidly from E18 to E27, and the weight of breast muscle was basically unchanged after E27. Through the expression profile analysis of DEGs, we obtained 8 trend maps (Fig. 3A). The expression of DEGs in trend 1 decreased from E18 to E27, and tended to be stable from E27 to E34 (Fig. 3B), which was opposite to the actual development trend of breast muscle. The expression of DEGs in trend 6 increased from E18 to E27, and tended to be stable from E27 to E34 (Fig. 3C), which was consistent with the actual breast muscle development trend. Therefore, we selected these two expression trends for further analysis.

**Fig. 4:**
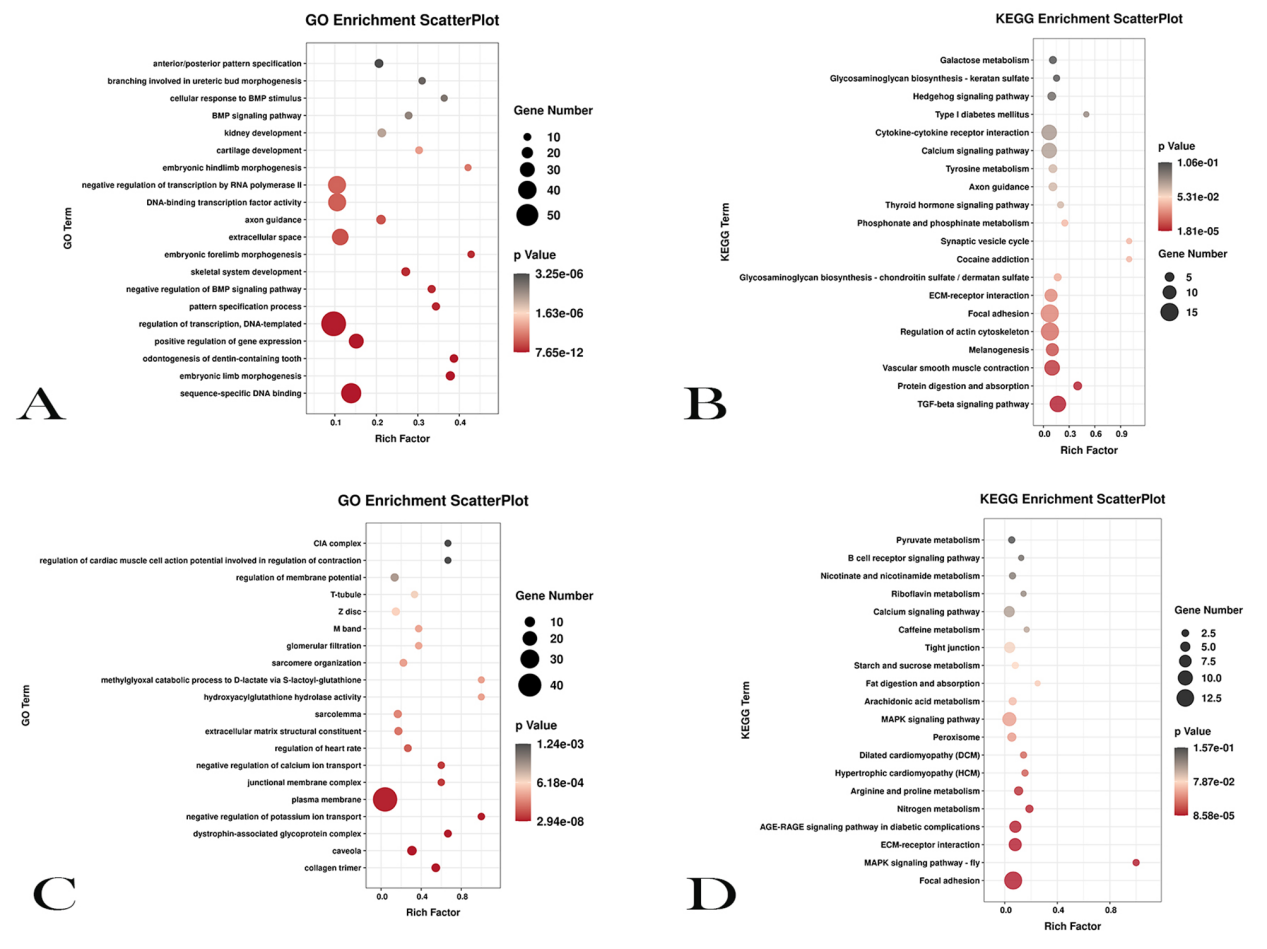
GO and KEGG analysis of transcriptome trend 1 and trend 6. (**A**) The top 20 GO biological processes enriched by DEGs in transcriptome trend 1. (**B**) The top 20 KEGG pathways enriched by DEGs in transcriptome trend 1. (**C**) The top 20 GO biological processes enriched by DEGs in transcriptome trend 6. (**D**) The top 20 KEGG pathways enriched by DEGs in transcriptome trend 6

There were 585 DEGs in trend 1 (Table S5). GO analysis of DEGs in trend 1 showed that most of the DEGs were significantly enriched in regulation of transcription, DNA templated, sequence specific DNA binding (*P* < 0.05, Fig. 4A and Table S6). KEGG analysis showed that DEGs were significantly enriched in TGF-beta signaling pathway, protein digestion and absorption, and vascular smooth muscle contraction. Among them, the TGF-beta signaling pathway was extremely significant (*P* < 0.01, Fig. 4B and Table S7), which was closely related to cell proliferation and differentiation. There were three genes, ID4, GDF5 and ID2, in this pathway, and their expression levels showed a downward trend during muscle development (Fig. 6).

There were 332 DEGs in trend 6 (Table S8). GO analysis of DEGs in trend 6 showed that most of the DEGs were significantly enriched in plasma membrane, extracellular matrix structural constituent, sarcolemma (*P* < 0.05, Fig. 4C and Table S9). KEGG analysis showed that the significantly enriched DEGs were related to MAPK signaling pathway, Focal adhesion, ECM − receptor interaction and other pathways (*P* < 0.05, Fig. 4D and Table S10).

### Joint analysis of transcriptomics and metabolomics

According to our preliminary experiments, we found a number of differential metabolites (DEMs) among E18, E27 and E34. Of them, the content of DEMs decreased from E18 to E27 and that of DEMs almost unchanged from E27 to E34 (Metabolome trend 1 and Table S11), which was particularly similar to the trend 1 of the DEGs analyzed above. There were also DEMs with their contents from E18 to E27, and were stable from E27 to E34 (Metabolome trend 6 and Table S12), which was basically consistent with the expression profiles of DEGs in transcriptome trend 6. Therefore, we conducted a joint analysis of the KEGG pathways for DEGs in transcriptome trend 1 and trend 6 and DEMs in the metabolome trend 1 and trend 6. By cross-comparing metabolomics and transcriptomics data, DEMs and DEGs were identified at the molecular and biochemical levels.

**Fig. 5:**
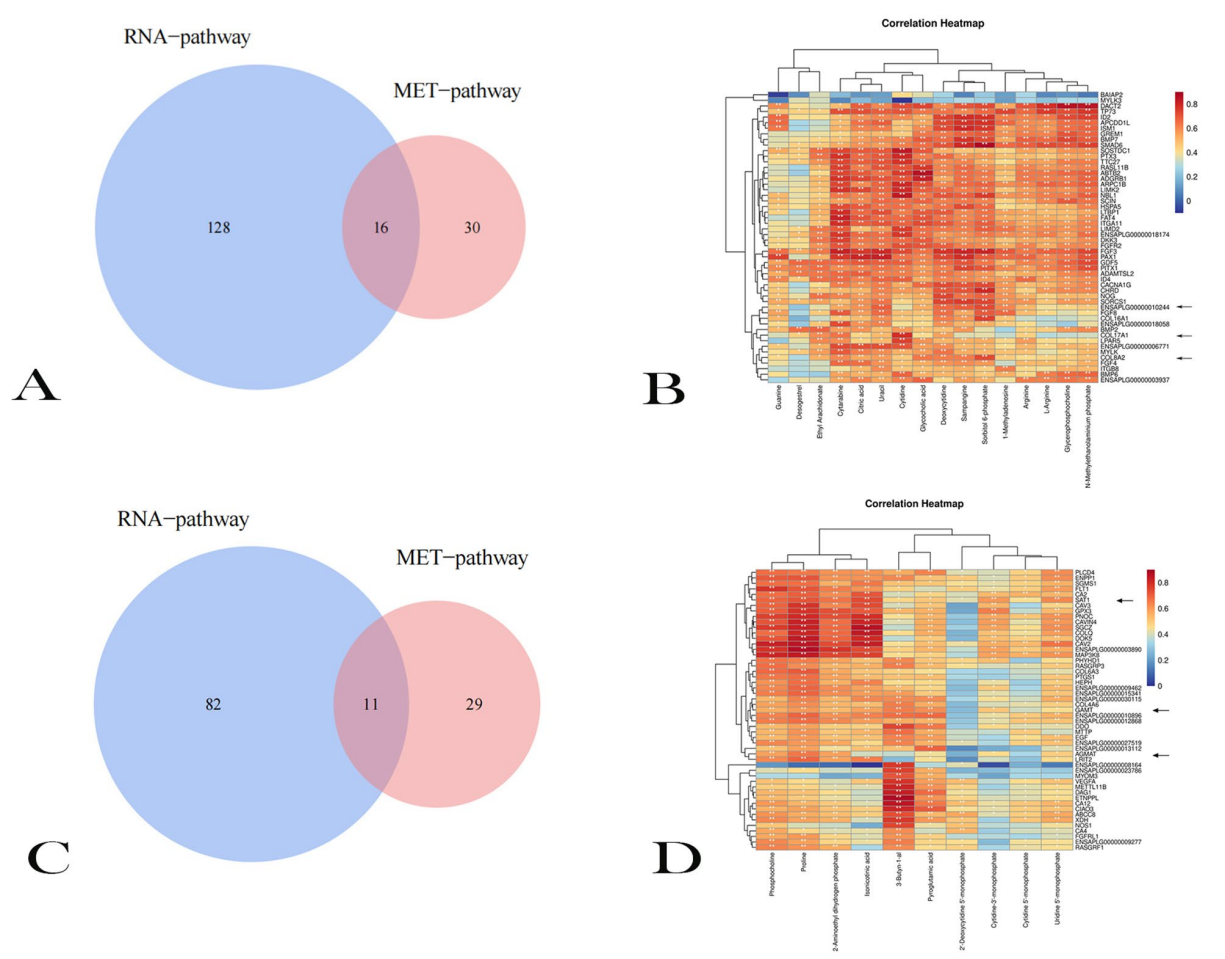
Joint analysis of transcriptome and metabolome. (**A**) The veen diagram of transcriptome trend 1 and metabolome trend 1. (**B**) The correlation between DEGs and DEMs in transcriptome trend 1 and metabolome trend 1. (**C**) The veen diagram of transcriptome trend 6 and metabolome trend 6. (**D**) The correlation between DEGs and DEMs in transcriptome trend 6 and metabolome trend 6

Between DEGs and DEMs in trend 1, 16 pathways were found to be shared by DEGs and DEMs, mainly including protein digestion and absorption, purine metabolism, and arginine biosynthesis (Fig. 5A). Protein is an important part of muscle. Genes that control protein synthesis may play important roles for muscle proliferation and differentiation. To this end, we further analyzed the protein digestion and absorption pathway, and explored DEGs and DEMs enriched in this pathway. The metabolites enriched in this pathway were arginine and L-arginine, DEGs included *COL17A1*, *COL8A2*, *COL16A1*, *ENSAPLG00000010244*.

The correlation between DEGs and DEMs was shown in Fig. 5B. From Fig. 5B, we can see that there was a significant positive correlation between arginine and *COL17A1, COL8A2, ENSAPLG00000010244*. A more in-depth analysis of these genes found that *COL8A2* has a tight relationship with cell proliferation [13].

In trend 6, there were 11 pathways shared by DEMs and DEGs, including sphingolipid metabolism, arginine and proline metabolism, pyrimidine metabolism, glutathione metabolism, etc. (Fig. 5C). Arginine and proline metabolism pathway played an important role in cell proliferation and differentiation [14, 15] and the metabolite enriched in this pathway was Proline. DEGs enriched in this pathway were *GAMT, NOS1, SAT1*, and *AGMAT*. From the correlation heatmap of genes and metabolites, a significant relationship between proline and *GAMT, SAT1, AGMAT* can be found (Fig. 5D).

## Discussion

Skeletal muscle development is mainly regulated by genes and influenced by endogenous metabolites. The identification of key genes and metabolites of skeletal muscle development in embryonic stage is of great useful in explaining the molecular mechanism of muscle development in meat ducks. A single-omics analysis only can not fully explain the relevant issues, so multi-omics analysis has been widely used [16, 17]. In this study, the transcriptome of Jiaji duck breast muscle during embryonic period was analyzed. The key genes and related metabolites of Jiaji duck breast muscle development during embryonic period were explained in this study. The results of this paper combined with our previous results of metabolomics analysis could provide a basis for the further study in exploring the mechanism of breast muscle development.

Through previous experiments, we found that the embryonic breast muscle cells proliferated rapidly and the weight of the breast muscle increased from E18 to E27. After E27, the cell proliferation almost stopped, and the weight of breast muscle did not change. The E34 was basically developed and ready for shelling. Therefore, we chose E18, E27, E34 to explore the key genes and metabolites that may be the regulators for skeletal muscle development of Jiaji ducks.

In this study, we found many DEGs in E18 VS E27, E27 VS E34, E18 VS E34 and conducted GO and KEGG pathway analysis for these DEGs. We obtained two pathways shared by E18 VS E27, E27 VS E34, E18 VS E34, ECM-receptor interaction and focal adhesion. ECM consists of a complex mixture of structural and functional macromolecules, mainly including collagen, fibronectin, and laminin [18, 19]. Studies have found that cell differentiation is affected by the interaction between cells and ECM [20, 21]. For focal adhesion, studies have shown the site where integrin and proteoglycan mediated adhesion connects with the actin cytoskeleton is called focal adhesion (FA), which is dynamic multi-protein complexes that connect ECM with the intracellular cytoskeleton [22, 23]. The formation and maturation of FA is a key procedure during myoblast differentiation. Thus, the GO and KEGG pathway analysis of DEGs of this study indicated that DEGs tightly correlated with the proliferation of breast muscle cell and the weight of breast muscle for Jiajia ducks.

In this study, DEGs in transcriptome trend 1 was contrast to the trend of breast development and myoblast cell proliferation. Thus, DEGs in transcriptome trend 1 may inhibit the development of embryonic breast muscle of Jiaji duck. KEGG analysis showed that TGF-beta signaling pathway was the extremely significant pathway, which was related to cell proliferation and differentiation [24]. DEGs in this pathway included *NOG, NBL1, CHRD, ID4, GREM1, GDF5, BMP2, BMP6, BMP7, SMAD6, LTBP1, ENSAPLG00000018058*, *ID2*. Among them, *ID4*, *ID2*, and *GDF5* were tightly related to skeletal muscle development. Study shows that *ID4* gene can inhibit cell differentiation [25]. In stem cells, *ID4* prevents other transcription factors from binding to DNA, thereby inhibiting the initiation of cell differentiation. This allows stem cells to continue to self-renew and proliferate, thereby maintaining their undifferentiated state [25]. Melnikova et al. found that *ID2* over-expression inhibited the differentiation of Sol8 myoblasts [26]. Sullivan et al. also showed that *ID2* inhibits cell differentiation but promotes the proliferation of different types of cells [27]. *GDF5* belongs to the TGF-β family and follows the same Smad-dependent and Smad-independent cell signaling pathways [28–31]. Moore et al. found that the addition of *GDF5* to the medium can lead to higher proliferation and ECM deposition of various cell types in a dose-dependent manner [29]. In summary, ID4, ID2 and GDF5 might be the key inhibitors of breast muscle development and myogenic cell proliferation.

We also found the expression profiles of DEGs in transcriptome trend 6 was consistent with the trend of breast muscle development and breast muscle cell proliferation of Jiaji ducks. Therefore, we supposed that DEGs in transcriptome trend 6 may promote the development of breast muscle and myoblast proliferation. In this study, we found DEGs were extremely significantly enriched in the MAPK signaling pathway, a pathway related to cell proliferation [32]. DEGs in this pathway included *LRIT2* and *DOK5*. *DOK5* protein can bind to a variety of growth factor receptors, such as EGFR and DGFR [33], and affect the transmission of growth factors, thereby affecting cell proliferation and differentiation. Xu et al. showed that over-expression of *DOK5* promoted cell proliferation and osteogenesis, and activated the canonical Wnt/β-catenin signaling pathway [34]. *LRIT2* with its expression level consistent with skeletal muscle development and myoblast proliferation of Jiaji duck may be a promotor for skeletal muscle development and myoblast proliferation. However, there is no report about the role of *LRIT2* up to date. Thus, the further study should be performed in the future. In addition, focal adhesion pathway, a pathway with extremely significant enrichment by DEGs in transcriptome trend 6, is closely related to cell proliferation and differentiation [23]. *EGF* is enriched in this pathway. Study shows *EGF* encodes epidermal growth factor [35], which is a signaling molecule that participates in the regulation of biological processes such as proliferation, differentiation by binding and activating EGF receptors on the cell surface [36]. Therefore, *DOK5, LRIT2* and *EGF* may be the crucial promoters of breast muscle development and myogenic cell proliferation.

**Fig. 6:**
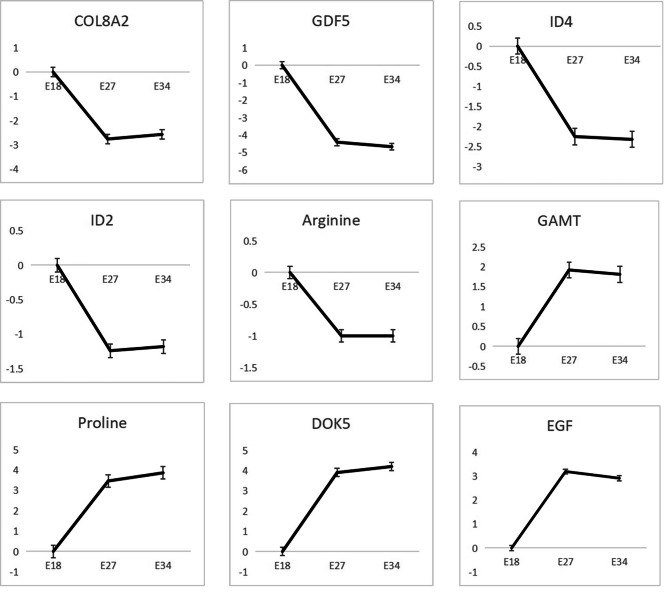
Relative changes of key genes and key metabolites in three stages of Jiaji ducks

Through the joint analysis, we found 16 overlapped pathways between transcriptome trend 1 and metabolome trend 1. Among them, the protein digestion and absorption pathway has been reported to be closely related to cell proliferation and differentiation [37, 38]. Further analysis found arginine and *COL8A2* were in the pathway. Arginine can be used as a synthetic substance and involve in the synthesis of protein and other cellular active substances [39]. Some studies have found that arginine regulates signal transduction of cell proliferation and differentiation by participating in mechanisms such as nitric oxide synthesis and release [40]. In addition, arginine is also involved in the synthesis of DNA and protein [41], which plays an important role in cell proliferation and differentiation. *COL8A2* involves in the construction and maintenance of extracellular matrix, and has a regulatory effect on cell proliferation and migration [13]. *COL8A2* is co-expressed with activating enhancer binding protein 2 (TFAP2) during corneal development, which has been reported to be related to the proliferation of CEC [42]. The co-expression of *COL8A2* and TFAP2 plays an important role in the migration and proliferation of vascular smooth muscle [43]. Hwang et al. found that the cell survival rate, cell proliferation rate, cyclin D1 expression and the number of cells in S phase decreased in cells transfected with *siCOL8A2* [13], indicating *COL8A2* has a inhibitory role for cell proliferation. In this study, the expression trend of *COL8A2* (Fig. 6) was contrast to the development trend of breast muscle of Jiaji duck, indicating that it had an inhibitory effect on the proliferation of muscle cells in the embryonic stage. In addition, a correlation analysis showed that arginine was significantly positively correlated with *COL8A2*, suggesting that the expression level of *COL8A2* has a tight linkage with the content of arginine. At the same time, this also shows that the decrease of *COL8A2* gene expression will lead to the decrease of arginine content, thus inhibiting the development of chest muscle of embryonic Jiaji ducks.

Through the joint analysis transcriptome trend 6 and metabolome trend 6, the arginine and proline metabolism pathway stood out because it was closely related to cell proliferation and differentiation. This pathway contains proline, which plays an important role in cell proliferation and differentiation [44] and can increase the rate of protein synthesis in muscle [15]. Proline can synthesize molecules related to protein synthesis, signal transduction and cell growth through the catalysis of a series of enzymes. Studies have shown that the regulation of the enzyme activity in the proline metabolic pathway can affect cell proliferation and differentiation [45]. DEGs enriched in this pathway are *GAMT*, *SAT1*, and *AGMAT*. The enzyme encoded by *GAMT* gene plays an important role in muscle cells [46], which is involved in the synthesis of creatine in muscle. Creatine directly affects the myogenic process (formation of muscle tissue), by altering secretions of myokines [47]. Arginine is the raw material for the synthesis of creatine [48]. There is a conversion relationship between proline and arginine [49–51], so proline has a certain effect on the synthesis of creatine. According to the correlation analysis, there is a significant positive correlation between *GAMT* and proline, so we believe that *GAMT* and proline jointly promote the proliferation of muscle cells.

## Conclusions

In summary, we can draw the following conclusions. The decrease of *COL8A2* gene expression will lead to the decrease of arginine content, which will inhibit the development of breast muscle in embryonic Jiaji duck. The increase of *GAMT* gene expression will cause the increase of proline content, so as to promote the development of breast muscle of Jiaji duck in embryonic period. In addition, *ID2*, *ID4* and *GDF5* genes may play a role in inhibiting muscle development during embryonic breast muscle development of Jiaji duck. *DOK5* and *EGF* genes may promote muscle development in this process.

### Electronic supplementary material

Below is the link to the electronic supplementary material.

Supplementary Material 1: Figure S1. Mapping of reads of breast muscle samples at different ages

Supplementary Material 2: Table S1. Mapping of reads from breast muscle samples of different stages to the reference genome

Supplementary Material 3: Table S2 E18VSE27_Gene_differential_expression

Supplementary Material 4: Table S3 E18VSE34_Gene_differential_expression

Supplementary Material 5: Table S4 E27VSE34_Gene_differential_expression

Supplementary Material 6: Table S5 Transcriptome trend 1 gene

Supplementary Material 7: Table S6 transcriptome trend 1 GO_enrichment_Gene

Supplementary Material 8: Table S7 transcriptome trend 1 KEGG_enrichment_Gene

Supplementary Material 9: Table S8 Transcriptome trend 6 gene

Supplementary Material 10: Table S9 transcriptome trend 6 GO_enrichment_Gene

Supplementary Material 11: Table S10 transcriptome trend 6 KEGG_enrichment_Gene

Supplementary Material 12: Table S11 Metabolomics trend 1 Metabolites

Supplementary Material 13: Table S12 Metabolomics trend 6 Metabolites

## Data Availability

All data generated or analysed during this study are included in this article and its additional files. All RNA-seq data analysed during this study has been deposited in NCBI database (https://submit.ncbi.nlm.nih.gov/subs/sra/) under accession number PRJNA1020383.

## Abbreviations

E18: 18th day after hatching
DEGs: differentially expressed genes
DEMs: differentially expressed metabolites
FA: focal adhesion
TFAP2: activating enhancer binding protein 2
