# Supplementary figures and images for: Integrated transcriptomics and metabolomics study of embryonic breast muscle of Jiaji ducks
Source: BMC Genomics. 2024 Jun 1;25:551. doi: 10.1186/s12864-024-10452-6 (PMC11144331; doi:10.1186/s12864-024-10452-6)

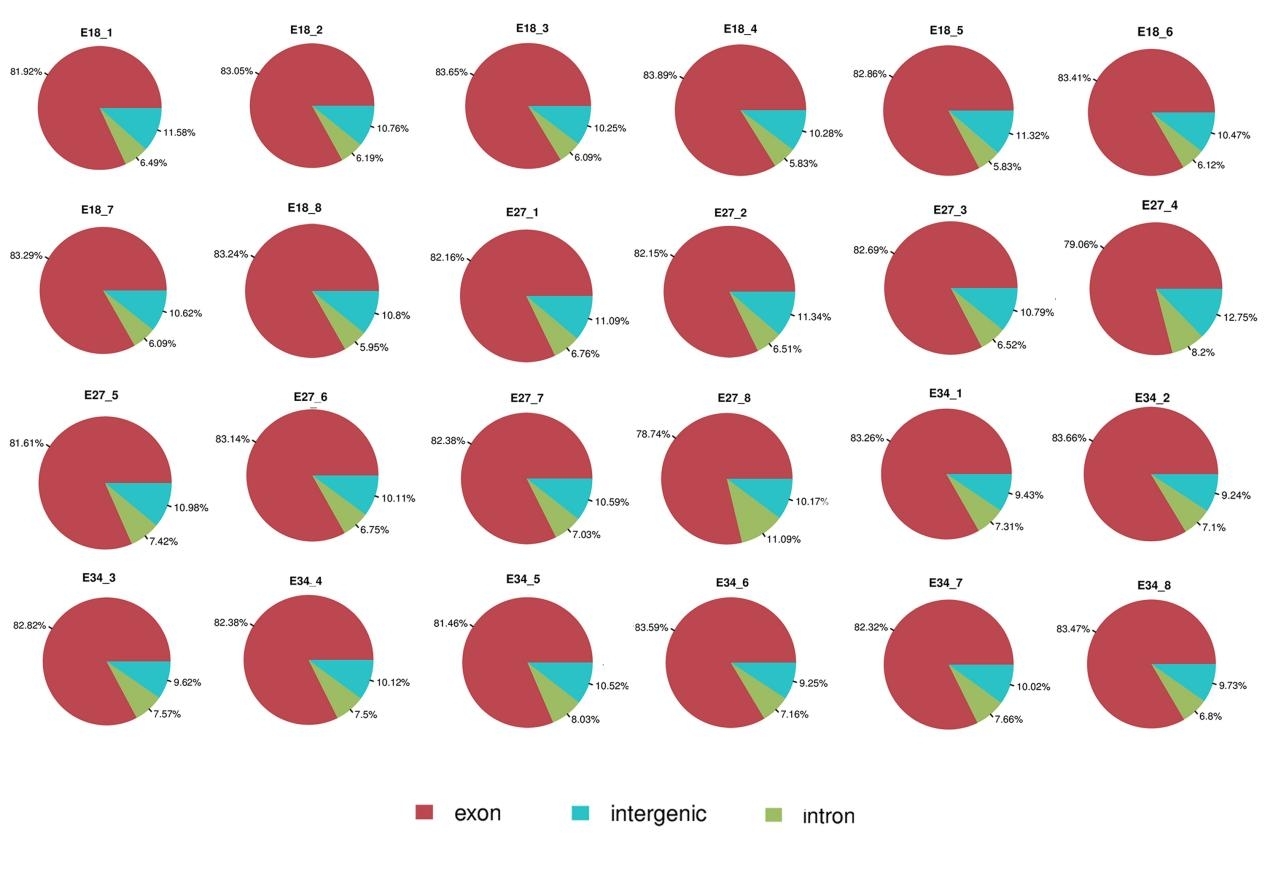


Figure S1. Mapping of reads of breast muscle samples at different ages

Supplement: Supplementary file 1 — Supplementary Material 1: Figure S1. Mapping of reads of breast muscle samples at different ages [file 12864_2024_10452_MOESM1_ESM.docx]
